# The role of economic stability in boosting exports in COMESA: Threshold effects analysis

**DOI:** 10.1371/journal.pone.0338636

**Published:** 2025-12-11

**Authors:** Amsalu K. Addis, Yang Xindong, Meng Tianze, Hailu Kendie Addis

**Affiliations:** 1 School of Economics and Management, Hanjiang Normal University, Shiyan, China; 2 Amhara Regional Agricultural Research Institute, Bahir Dar, Ethiopia; United Arab Emirates University, UNITED ARAB EMIRATES

## Abstract

Economic stability is crucial for improving export performance, particularly within regional trade blocs such as the Common Market for Eastern and Southern Africa (COMESA). However, empirical studies examining how threshold levels of economic stability affect export dynamics among COMESA member states have been limited. This study explores the threshold effects of key economic indicators, economic growth, trade openness, and inflation, on export performance in COMESA countries using panel data from 2000 to 2022. Employing panel threshold regression analysis, this study investigates the nonlinear relationships between these variables, focusing particularly on the impact of inflation thresholds. The study found an optimal inflation threshold of 3.662%, aligning with the lower end of the global benchmark of 3–7% typically observed in emerging and developing economies. In low-inflation regimes (LnInfl ≤ 3.662), GDP significantly enhances export growth by 0.348% per unit increase, while trade openness yields a smaller contribution of 0.091%. In contrast, high-inflation scenarios (LnInfl > 3.662) show that GDP continues to support exports (0.779% increase), but trade openness negatively impacts export performance by 0.127%. Policymakers need to focus on maintaining inflation under the critical threshold of 3.662%, as this is vital for enhancing the benefits of GDP growth and trade openness on export performance. These insights underscore the necessity of keeping inflation in check to improve export competitiveness. Additionally, prioritizing monetary, fiscal, and institutional stability initiatives will support sustainable growth and strengthen regional trade dynamics within COMESA, as exceeding this inflation limit could impede export potential and regional integration efforts. This study offers valuable insights for developing regions aiming to enhance their trade performance through effective economic policies.

## 1. Introduction

Economic stability involves moderate inflation, consistent GDP growth, minimal exchange rate volatility, sustainable fiscal deficits, and a conducive environment for trade [1–3]. This stability is essential for fostering investor confidence and enhancing export performance, particularly within regional trade blocs such as the Common Market for Eastern and Southern Africa (COMESA) [4,5]. The study of the complex relationship between economic stability and export growth has emerged as a critical area within international trade and development economics. This relationship is of paramount importance, as it can significantly influence the economic trajectories of member countries, particularly in developing regions. Economic stability, characterized by factors such as controlled inflation, robust GDP growth, and trade openness, plays a pivotal role in shaping export performance [3,6,7]. For instance, inflation, has been identified as a key determinant of macroeconomic stability, influencing both domestic production costs and international competitiveness. Understanding how inflation interacts with other economic variables to affect export growth is essential for designing effective policies aimed at fostering sustainable development [8,9].

In the context of the COMESA, where member states exhibit diverse levels of economic development and varying degrees of macroeconomic stability, analyzing these dynamics becomes even more critical [10,11]. Inflationary pressures can create nonlinear effects on export performance by altering the relative impact of other factors such as GDP growth and trade openness. For instance, low-inflation may enhance the positive effects of GDP growth on exports by maintaining cost competitiveness, while high-inflation could negate these benefits by increasing uncertainty and discouraging investment [8,9]. Investigating these threshold effects is critical for understanding the dynamics of export growth across member states.

COMESA consists of 21 member countries and serves as a regional economic community dedicated to promoting economic integration and cooperation among its members. The export performance of this region is influenced by a variety of macroeconomic factors, including GDP growth, trade openness, and inflation levels. The relationship between macroeconomic factors and export performance is often nonlinear and complex. For example, trade openness has been identified as a significant driver of export performance, facilitating Foreign Direct Investment (FDI) inflows that can bolster a country’s export capabilities [12–17]. Similarly, GDP growth serves as an indicator of a country’s productive capacity and its competitiveness in international markets [8,18].

Inflation influences export dynamics in dual ways; it can either stimulate or deter exports depending on its levels. Moderate inflation can increase competitiveness by making domestic goods relatively cheaper in global markets, while high-inflation may discourage investment and diminish export competitiveness [9,19,20]. Stable inflation helps maintain purchasing power and lowers production costs, fostering economic activity. However, excessive inflation may destabilize the economic, eroding competitiveness and hindering trade opportunities [21].

Economic stability is of paramount importance for enhancing export performance, particularly within regional economic frameworks like COMESA [1–3]. Although FDI has been acknowledged as a catalyst for export growth [22], contemporary literature underscores the significance of macroeconomic determinants, including inflation rates, GDP growth, and trade openness, in shaping export competitiveness [23]. A stable economic environment not only attracts investment but also enhances the competitiveness of domestic on a global scale by alleviating uncertainty and promoting operational efficiency. In particular, inflation exerts a considerable influence on economic stability, with its impact on export growth demonstrating a nonlinear characteristic and being associated with threshold effects. A comprehensive understanding of these macroeconomic interactions is essential for formulating productive export strategies within the COMESA region [24,25].

Scholars and policymakers have recognized the profound transformative impacts of economic stability on export levels and sustainable economic growth [26]. While existing literature has extensively explored the impact of GDP growth, trade openness, and inflation on economic performance across various regions [3,5], there is a notable research gap regarding the specific impact of economic stability on export growth within the COMESA framework. In particular, limited attention has been given to understanding the nonlinear effects of inflation as a threshold variable that may moderate the relationship between GDP growth, inflation rate, trade openness, and export performance.

This study conducts the effect of economic stability, particularly inflation rate, on export growth in 12 COMESA countries from 2000 to 2022. Utilizing a Panel Threshold Regression (PTR) model [27], the study examines whether inflation acts as a threshold variable that segregates the relationship between export growth and other explanatory factors such as GDP growth and trade openness into distinct regimes. By identifying these thresholds, the study sheds light on how varying inflation levels influence these determinants of export performance.

The principal uniqueness and contributions of this study encompass: *First*, the elucidation of nonlinear dynamics that demonstrate how the repercussions of inflation on export growth may fluctuate according to varying conditions. This understanding is crucial for policymakers who seek to comprehend the impacts of inflation on export growth across diverse economic contexts. *Second*, the investigation of specific low- and high-inflation regimes yields pragmatic insights for government entities to formulate targeted economic policies, such as the stabilizations of inflation levels to optimize the positive effects of trade openness on export performance. *Third*, the relevance of this study is particularly pronounced for the member states of COMESA, considering their heterogeneous economic frameworks, thereby underscoring the necessity of optimal conditions for the enhancement of export activities.

This study aims to furnish policymakers and pertinent stakeholders with essential insights regarding the intricate interplay between economic stability and export performance within COMESA. By fostering a more profound comprehension of these complex dynamics, it aspires to guide strategic policy measures that uphold optimal inflation rates, thereby maximizing the advantages derived from GDP growth and trade liberalization for the purpose of export growth. Importantly, this study addresses a significant gap in the existing literature by demonstrating how nonlinear interactions among macroeconomic variables affect trade outcomes in developing regions such as COMESA. As the premier analysis of the correlation between economic stability and export expansion in this specific region, it enriches our understanding of the interplay between inflation, GDP, and trade openness, and how these factors collectively influence export growth and facilitate economic growth. Employing a PTR framework, the present study elucidates the nonlinear effect of inflation on export expansion, revealing the extent to which varying inflation rates influence the impacts of GDP growth and trade liberalization. Through an analysis of the interaction between threshold inflation levels and these macroeconomic factors, the study provides policy recommendations designed to bolster export performance while concurrently fostering inclusive growth, advancing market integration, and promoting sustainable development.

Furthermore, this study addresses several pivotal questions: How do export growth, inflation, GDP, and trade openness interact across COMESA countries? What inflation thresholds significantly alter these relationships? What export growth trends have emerged from 2000 to 2022? Which countries have demonstrated consistent export growth under stable economic conditions, and what factors contribute to this consistency? How do varying levels of trade openness interact with inflation thresholds to influence export performance? By methodologically addressing these inquiries through rigorous econometric modeling and empirical analysis, this study proffers invaluable policy recommendations aimed at fostering sustainable, export-led growth in COMESA economies.

## 2. Literature review

The relationship between export growth and macroeconomic variables such as GDP, inflation, and trade openness is a significant area of study in international economics. Several studies have explored these relationships across different countries and time periods [23,28–32]. A thorough and meticulous examination of the existing body of literature uncovers a plethora of varying viewpoints regarding the intricate and complex relationships that exist among economic stability, the growth of exports, the overall economic expansion as measured by GDP, the phenomenon of inflation, and the degree of trade openness within different economies. The fundamental assertion underlying this discourse posits that a macroeconomic environment characterized by stability serves to diminish levels of uncertainty, thereby fostering an atmosphere conducive to investment, while simultaneously augmenting the competitive edge of businesses, which collectively contribute to the stimulation of activities that are primarily focused on exportation [33,34].

The interplay between economic stability and export performance is integral to understanding the dynamics of international trade. Economic growth, as measured by GDP, significantly influences a nation’s export capacity. Elevated economic growth can lead to enhanced production levels and an expanded supply of goods for export [35]. However, rapid GDP growth may also trigger inflationary pressures and an appreciation of the domestic currency, ultimately diminishing export competitiveness [36]. Trade openness, typically quantified as the ratio of total trade to GDP, acts as a critical determinant of a country’s integration into the global market. Increased trade openness can facilitate access to broader markets, foster specialization, and optimize efficiency, thereby augmenting export performance [37]. Nonetheless, the benefits of trade openness tend to vary with a country’s developmental stage; less developed economies may encounter adverse effects when trade openness is below a certain threshold, while more developed nations often reap positive returns [38]. Additionally, inflation plays a pivotal role in shaping export dynamics; high inflation can inflate production costs, erode price competitiveness, and instigate currency depreciation, an impetus that, while potentially enhancing export volumes in the short term, may also breed uncertainty and deter investment [39]. Therefore, effective inflation management becomes essential for sustaining stable export growth [36].

The complexity of the relationship between these economic indicators and export performance is further augmented by threshold effects, where the impact of an independent variable on exports transforms at specific levels. For instance, panel threshold regression analyses reveal a nonlinear relationship between trade openness and economic growth, indicating that its positive effects materialize only after surpassing a developmental threshold [38]. Similarly, the influence of economic growth on export performance may fluctuate based on the degree of trade openness; for countries with varying levels of engagement in international trade, the correlation between export volume and GDP growth may differ significantly [40]. Furthermore, the interaction between infrastructure development and trade openness is crucial, as enhanced infrastructure can amplify the positive effects of trade openness on a nation’s economic transformation [37].

In the context of the COMESA region, efforts to stimulate sustainable economic growth through increased regional trade have not consistently yielded significant positive outcomes. While COMESA seeks to foster integration among member states, the effectiveness of these initiatives is influenced by various factors, including infrastructure quality, trade policies, and institutional integrity [37,41]. Panel data analysis emerges as a powerful statistical approach for investigating the relationships between these variables across nations over time, allowing for robust controls of heterogeneity among entities and dynamic interactions [42].

Moreover, external factors such as global economic conditions, trade policies of major trading partners, and fluctuations in commodity prices critically influence export performance within regions like COMESA countries [43,44]. Institutional quality, including efficient customs procedures, contract enforcement, and property rights protections, is vital for bolstering trade and attracting investment [45]. Political stability and a lack of violence are also essential for creating a conducive environment for economic growth and trade [45]. Overall, the ease of doing business, which encompasses aspects like the simplicity of starting a business and cross-border trade, significantly impacts economic growth and trade dynamics [46]. Additionally, the efficiency of seaports is a critical factor that enhances the positive effects of trade on economic welfare, highlighting the interconnectedness of trade facilitation and economic outcomes [47]. By examining these complex relationships, researchers can develop a comprehensive understanding of the economic factors that drive export performance and stability within and beyond the COMESA region.

## 3. Methodology

### 3.1. Dataset and sources

This study examines how economic stability, particularly inflation thresholds, affects export growth rates among 12 COMESA countries. We gathered annual panel data from 2000 to 2022, focusing on variables that are grounded in economic theories and empirical studies on international trade and macroeconomic stability, for instance [27,48–51]. The selection of variables was guided by their relevance to the research question and their role in explaining export performance under varying inflationary conditions, as well as their availability across COMESA countries. Missing data were handled using linear interpolation methods to ensure continuity without introducing bias. The number of interpolated gaps totaled nearly a dozen from over 275 observations.

Key macroeconomic factors influencing export growth include GDP, trade openness, and inflation, with export growth serving as the dependent variable. Inflation is treated as the threshold variable to capture its nonlinear effects on export growth. The choice of inflation as the threshold variable in analyzing the role of economic stability in boosting exports in COMESA, particularly within a Panel Threshold Regression (PTR) model, is theoretically sound and empirically supported by various economic principles [52,53]. Inflation, as a key indicator of macroeconomic stability, directly impacts several channels relevant to export performance.

The nexus between export growth and key macroeconomic variables such as GDP, inflation, and trade openness represent a crucial focus in the field of international economics. A number of studies have examined these relationships across various countries and timeframes [23,28–32,49,54,55]. These investigations provide valuable insights into how these macroeconomic factors interact and influence export performance globally. The data for these variables were sourced from reliable and reputable databases such as the World Bank’s World Development Indicators (WDI) and Our World in Data (OWID). These comprehensive and standardized datasets are widely recognized and facilitate cross-country comparisons over time, as supported by various studies utilizing similar datasets [56]. Details of the variables and sources used are provided in Table 1.

**Table 1:** Variables and data sources.

Variables	Symbols	Measurement units	Availability	Data source
Economic growth	LnGDP	Annual %	2000-2022	WDI (2023)
Export Growth	LnEG	Exports of goods and services (annual % growth)	2000-2022	WDI (2023)
Inflation rate	LnInfl	Consumer prices (annual %)	2000-2022	WDI (2023)
Trade Openness	LnTO	Trade (% of GDP)	2000-2022	OWID (2023)

All variables were transformed into natural logarithms (Ln).

In this study, annual export growth rates serve as the dependent variable, reflecting the ability of COMESA countries to expand their trade activities over time. This metric directly measures the effectiveness of these nations in international trade. To evaluate the impact of economic factors, we include GDP as an indicator of economic size and overall productivity, which is essential for a country’s capacity to produce exportable goods. GDP provides insight into domestic production capabilities, crucial for understanding supply-side influences on exports. Trade openness is assessed through the ratio of total trade (exports plus imports) to GDP, illustrating a country’s integration into global markets. This variable reflects policy initiatives aimed at enhancing global economic participation, which can attract foreign investment and boost export performance. Inflation rates are included as both an explanatory and threshold variable to explore their nonlinear effects on export growth. Inflation plays a dual role by influencing competitiveness and economic stability. By treating inflation as a threshold variable, the study seeks to identify specific levels at which inflation begins to either hinder or enhance export performance. Table 2 lists the 12 COMESA member countries included in this study. These countries were chosen solely due to data availability.

**Table 2:** List of COMESA member countries used in the study analysis.

Burundi	Eswatini	Mauritius	Sudan
Comoros	Kenya	Rwanda	Tunisia
Egypt	Madagascar	Seychelles	Uganda

The remaining COMESA member countries were excluded from the study due to a lack of available data (refer to S1 Table in the Supporting information section for detailed information).

### 3.2. Methodological framework

**Fig 1:**
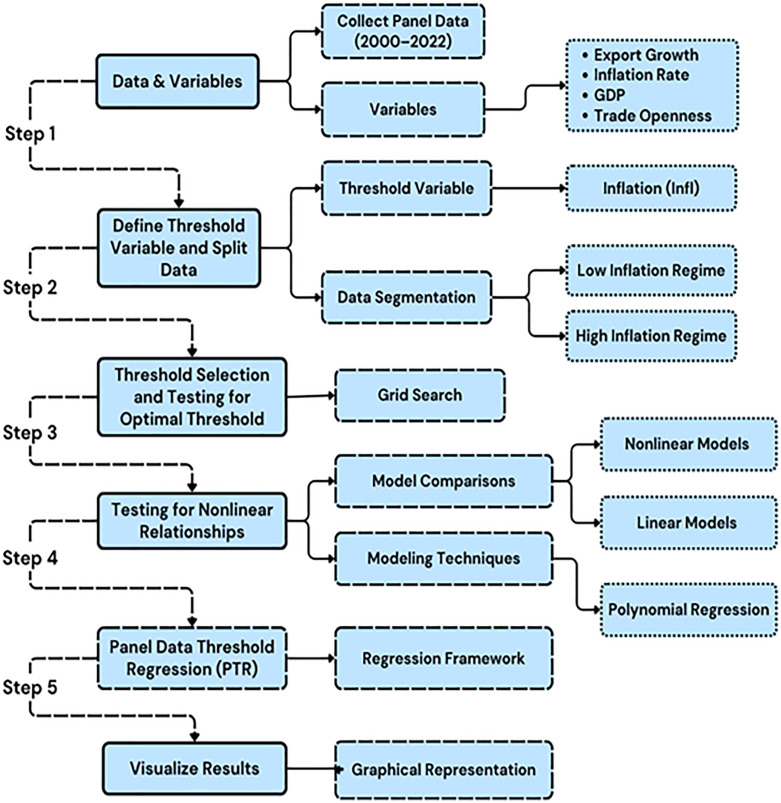
Methodological framework for analyzing threshold effects of inflation on export growth in COMESA.

The methodological framework for this investigation, as depicted in Fig 1, is systematically organized around a series of pivotal steps aimed at examining the repercussions of inflation on export expansion through the implementation of threshold regression analysis. First, the study defined/selected inflation (Infl) as the threshold variable, and the dataset is divided into two distinct regimes—low inflation and high inflation—based on a predetermined threshold value. Next, a grid search is conducted to determine the optimal threshold by evaluating various values of Infl and minimizing the residual sum of squares (RSS). Grid search is a recognized approach for assessing the robustness of threshold parameters [57]. Subsequent to this, the investigation of nonlinear relationships is conducted to ascertain the presence of threshold effects; this necessitates the evaluation of whether nonlinear models yield a better fit relative to linear models, employing techniques such as polynomial regression, particularly quadratic regression, to elucidate potential complexities [58,59]. The analysis subsequently advances to a Panel Data Threshold Regression (PTR), as delineated by [27], which is employed in this study to assess the interactions among inflation, GDP, and trade openness to understand their combined effects on export growth. Finally, the findings are represented visually by graphing export growth against inflation, incorporating fitted lines for both the low and high inflation regimes, thus facilitating a more lucid interpretation of the influence of inflation on export dynamics across varying economic contexts. This thorough approach guarantees a rigorous analysis of the threshold effects of inflation on exports within the COMESA region.

### 3.3. Data analysis

The research framework of this study is to investigate whether inflation acts as a threshold variable that moderates the relationship between export growth and key economic factors such as GDP and trade openness in COMESA countries. The Panel Threshold Regression (PTR) model, introduced by Hansen (1999), extends the linear panel data regression model to account for nonlinear threshold effects. Threshold effects analysis is an econometric technique that identifies critical values (thresholds) of an independent variable, at which the relationships between other variables change significantly [1,48,60]. The PTR model is an analytical tool used to investigate nonlinear relationships between variables, particularly when these relationships change as a result of another variable exceeding a specific threshold [61,62]. A nonlinear relationship is characterized by the phenomenon wherein the influence of one variable on another does not exhibit a consistent change; rather, the nature of this relationship may fluctuate across varying levels of the independent variable. For example, within the framework of export growth, the impact of GDP on export levels may escalate at an increasing rate after surpassing a certain GDP threshold, suggesting that as national economies experience growth, their ability to augment exports may become more evident, albeit not uniformly across differing GDP magnitudes.

The PTR approach is frequently applied in economic and financial research to identify regime-switching behaviors and structural breaks within data [63,64]. The PTR model is particularly well-suited for addressing heterogeneity; it enables the interaction between independent and dependent variables to differ across various regimes established by the threshold variable [65]. Therefore, to identify nonlinear relationships and estimate threshold effects of inflation on export growth, our study employed a PTR model. This nonlinearity arises because the relationship between the dependent variable and independent variables change depending on whether the threshold variable crosses a specific value.

It is essential to introduce the equation of linear relationships before transitioning to nonlinear relationships and estimating threshold effects in our study. To ensure consistency and comparability across countries, all variables were transformed into natural logarithms. This transformation helps to stabilize variance, reduce skewness, and interpret coefficients as elasticities in regression models. For this study, using Export Growth (EG) as the dependent variable and economic growth (GDP) and Trade Openness (TO) as independent variables, the linear panel data regression model can be expressed as:


$$LnEG_{it}=\alpha_{1}+\beta_{1}LnGDP_{it}+\gamma_{1}LnTO_{it}+\varepsilon_{it}$$ (1)

Where, Ln stands for the natural logarithm, $EG_{it}$ refers to export growth for country i at time t, $GDP_{it}$ represents economic growth for country i at time t, and $TO_{it}$ implies trade openness for country i at time t. Here α is the intercept term, while β_1_ and γ_1_ are coefficients representing the effect of each independent variable on export growth, and $\varepsilon_{it}$ is the error term capturing unobserved factors affecting export growth. This equation assumes a linear relationship between export growth and its determinants across all countries and time periods in the dataset, with no threshold effect or nonlinearity in the relationship between these variables.

Unlike linear models where coefficients remain constant across all observations, PTR models allow for different coefficients in different regimes. The PTR model is widely used in economic studies to capture regime changes or structural breaks caused by specific threshold levels. Taking inflation (LnInfl) as the threshold variable, we can write the PTR model explicitly as:


$$\begin{array}{c} LnEG_{it}=\{\alpha_{1}+\beta_{1}LnGDP_{it}+\gamma_{1}LnTO_{it}+\varepsilon_{it},\;if\;LnInfl_{it}\leq c\;\alpha_{2}+\beta_{2}LnGDP_{it}+\gamma_{2}LnTO_{it}+\varepsilon_{it},\;if\;LnInfl_{it}>c \end{array}$$ (2)

Here, $Infl_{it}$ denotes inflation (a threshold variable) for country i at time t, (c > 0: Threshold value that splits the data into two regimes. This equation shows that when inflation (LnInfl) is below or equal to a certain threshold level (c), one set of coefficients applies (α_1_, β_1_, γ_1_). When inflation exceeds this threshold level, another set of coefficients applies (α_2_, β_2_, γ_2_).

The PTR model allows for nonlinear relationships by introducing threshold effects. The decision to use PTR instead of linear regression ones in this study is based on the nature of the economic phenomena being analyzed. Export growth, inflation, GDP, and trade openness often exhibit complex interactions that cannot be adequately captured by simple linear models. Employing PTR allows the study to: (i) Capture threshold effects where key variables like inflation influence export growth differently across distinct regimes. (ii) Provide more accurate empirical evidence consistent with economic theory and real-world observations. (iii) Improve model fit and predictive power by accounting for heterogeneity among member states. (iv) Offer nuanced policy recommendations tailored to specific conditions within COMESA countries.

In economic studies, relationships between variables are often assumed to be linear for simplicity. However, testing for nonlinear relationship is crucial because it allows researchers to capture the complex interactions among variables and better understand the true nature of the relationships. For instance, in this study, export growth may not respond uniformly to changes in inflation, GDP, or trade openness. Threshold effects, where the impact of one variable changes depending on the level of another variable, are a common form of nonlinearity.

By conducting nonlinear relationship testing, such as fitting quadratic regression models or incorporating interaction terms with threshold variables (inflation regimes), we can identify whether these nonlinearities exist and how they influence the dependent variable. This approach ensures that policy recommendations derived from the analysis are more accurate and reflective of real-world dynamics. Without testing for nonlinearity, important insights could be missed, leading to biased or oversimplified conclusions and potentially ineffective or counterproductive policy measures. The equations for nonlinear relationship testing used in the study can be expressed as:

1. Linear model (Model 1),

$$LnEG=\beta_{0}+\beta_{1}LnInfl+\beta_{2}LnGDP+\beta_{3}LnTO+\varepsilon$$ (3)

2. Quadratic model (Model 2),

$$LnEG=\beta_{0}+\beta_{1}LnInfl+\beta_{2}(LnInfl{)}^{2}+\beta_{3}LnGDP+\beta_{4}LnTO+\varepsilon$$ (4)

Where, the additional term $(LnInfl{)}^{2}$ captures the nonlinearity in the relationship between inflation and export growth.

3. Interaction model with threshold effects

The interaction model incorporates a dummy variable for inflation regimes (LowRegime = 1, HighRegime = 0) and interaction terms between explanatory variables and the regime. The equation is:


$$\begin{array}{cc} LnEG=\; & \beta_{0}+\beta_{1}(LowRegime)+\beta_{2}(LnGDP)+\beta_{3}(LowRegime:LnGDP)\\ & \;\;+\;\beta_{4}(LnTO)+\beta_{5}(LowRegime:LnTO)+\varepsilon \end{array}$$ (5)

Where. LowRegime represents a dummy variable for inflation regimes, β_0_: Intercept, β_1_: Effect of GDP on export growth, β_2_: Effect of being in a low-inflation regime, β_3_: Effect of trade openness on export growth, β_4_: Direct effect of inflation, though not statistically significant, β_5_: Interaction effect between GDP and low-inflation regime, and β_6_: Interaction effect between trade openness and low-inflation regime.

Furthermore, to identify whether the effect of one independent variable on the dependent variable changes depending on the level of another independent variable and to improve the explanatory power of the model by accounting for complex interdependencies among variables that would otherwise be overlooked in a simple additive model, this study employed interaction terms in a regression model. Testing interaction terms in a regression model is crucial for understanding how two or more variables jointly influence the dependent variable. In this case, testing the interaction terms between inflation and other variables (GDP and trade openness) provides insights into how inflation moderates or amplifies their effects on export growth. It provides a deeper understanding of how multiple factors work together to influence export growth, enabling more effective policymaking and economic planning. The regression model with interaction terms can be expressed as follows:


$$\begin{array}{cc} LnEG=\; & \beta_{0}+\beta_{1}LnGDP+\beta_{2}LnTO+\beta_{3}LnInfl+\beta_{4}(LnInfl\cdot LnGDP)\\ & \;\;+\;\beta_{5}(LnInfl\cdot LnTO)+\varepsilon \end{array}$$ (6)

Where, β_0_,...,β_5_: Coefficients estimated by the regression, (LnInfl⋅LnGDP) = Infl_GDP: Interaction term between inflation and GDP, and (LnInfl⋅LnTO) = Infl_TO: Interaction term between inflation and trade openness.

## 4. Results and discussion

### 4.1. Descriptive statistics for low- and high-inflation regimes

This study investigates the impact of economic stability on export growth within the COMESA region, specifically examining threshold effects using inflation as the threshold variable. By splitting the data into low inflation and high inflation regimes based on the median inflation value, we gain insights into the economic dynamics across these two regimes from 2000 to 2022. The summary statistics, presented in the Table 3, outline key metrics including export growth (LnEG), GDP (LnGDP), trade openness (LnTO), and inflation (LnInfl) for both regimes.

**Table 3:** Descriptive statistics for low-and high-inflation regimes.

Low-inflation regime						
Variable	Minimum	1st Quartile	Median	Mean	3rd Quartile	Maximum
Year	2000	2006	2013	2012	2018	2022
LnEG	3.953	4.578	4.630	4.626	4.689	4.985
LnGDP	1.696	3.109	3.171	3.163	3.242	3.502
LnTO	3.164	3.682	4.012	4.173	4.634	5.393
LnInfl	0.572	2.129	2.315	2.225	2.441	2.562
**High-inflation regime**						
**Variable**	**Minimum**	**1st Quartile**	**Median**	**Mean**	**3rd Quartile**	**Maximum**
Year	2000	2005	2009	2010	2015	2022
LnEG	0.492	4.565	4.642	4.625	4.741	5.215
LnGDP	1.097	3.081	3.176	3.126	3.239	3.439
LnTO	0.693	3.608	3.890	3.845	4.224	5.425
LnInfl	2.565	2.679	2.833	2.986	3.114	5.491

The result provides insights into the economic dynamics of COMESA countries under low- and high-inflation conditions. In terms of export growth, both inflation regimes presented almost identical mean values, around 4.625 to 4.626. However, the low inflation regime exhibited a wider range, with a maximum export growth of 4.985 compared to 5.215 in the high inflation group. This suggests that economies with lower inflation may experience more stable trading conditions, potentially leading to higher export growth and resilience against economic shocks [36]. Elevated inflation rates can result in the depreciation of a currency, potentially providing a short-term increase in exports. However, this scenario often generates economic uncertainty, which can deter investment [39]. On the other hand, low and stable inflation is typically viewed as beneficial for the economy, fostering an environment conducive to stability, investment, and growth. Nonetheless, maintaining inflation at excessively low levels is also critical, as it can pose its own set of economic challenges and risks [66].

Economic growth showed notable differences between the two regimes, particularly in the lower bounds. The minimum LnGDP growth was 1.696 in the low inflation group, contrasting sharply with the high inflation group’s minimum of 1.097. This indicates that countries in low inflation environments tend to have a stronger baseline for economic growth, while those facing higher inflation may encounter challenges that hinder robust economic performance. Notably, the mean LnGDP growth values were slightly higher in the high inflation regime, at approximately 3.126, compared to 3.163 in the low inflation context. This indicates the possibility that high inflation regions can still generate significant growth, albeit with greater risk.

When examining trade openness, the data revealed that the average openness was significantly higher in the low inflation regime at 4.173 versus 3.845 in the high inflation regime. This implies that countries maintaining low inflation may enjoy more favorable conditions for trade. Furthermore, although both regimes exhibited high maximum values for trade openness, the lower bound in the high inflation grouping (0.693) suggests that some countries are considerably less open to trade due to the economic pressures stemming from inflation. The inflation variable clearly distinguished the two groups, with the low inflation regime reporting a mean of 2.225, while the high inflation regime averaged 2.986. The broader range of inflation values in the high inflation context, where the minimum was 2.565 and the maximum reached 5.491, highlights the economic fragility encountered by these countries.

The analysis indicates that countries experiencing high inflation should emphasize policies aimed at stabilizing inflation, given its negative correlation with GDP growth and trade openness [36,66]. High inflation can erode competitiveness by increasing production costs and reducing profit margins for exporters. Conversely, maintaining lower inflation appears beneficial for sustaining higher export growth and economic resilience [66]. Therefore, further investigation into sector-specific performances, alongside comprehensive regression analyses, could yield valuable insights, particularly regarding the relationship between inflation and economic outcomes. This approach may assist policymakers in implementing strategies that promote economic stability and growth, tailored to the unique challenges posed by varying inflation levels.

**Fig 2:**
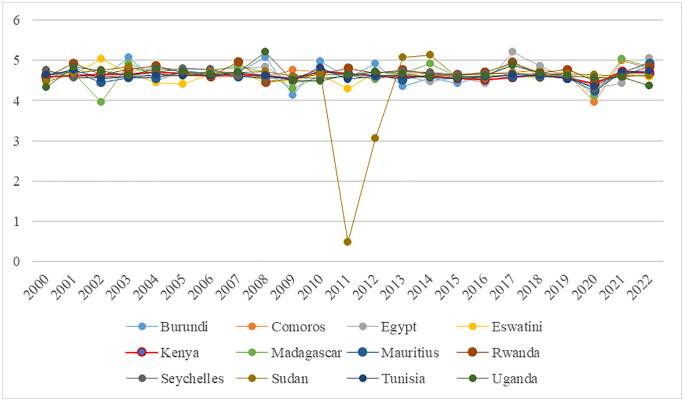
Trends in export growth for COMESA countries, spanning 2000–2022.

The average export value in the COMESA region is approximately 4.63, which aligns with its median, suggesting a fairly balanced distribution. When analyzing individual countries export performance, significant variability exists, with export values ranging from a minimum of 0.49 to a maximum of 5.22 (see Fig 2). Most countries show relatively stable export growth with only minor fluctuations, although there was a notable decline around 2011, likely due to economic or external factors impacting exports during that time. Kenya stands out with the most consistent export growth among the analyzed countries, marked by a low standard deviation of 0.072. This stability highlights the crucial role of trade openness and inflation rates in shaping export performance. Political stability and governance, human capital, trade agreements and policies, and infrastructure development create a resilient and adaptive export environment that enables consistent growth.

### 4.2. Threshold effect analysis and testing for optimal threshold

Economic relationships are frequently nonlinear, meaning that the impact of one variable on another can change once a certain level or “threshold” is crossed [67,68]. The threshold effect analysis seeks to reveal nonlinear patterns that traditional linear regression approaches might miss, thereby providing a more nuanced understanding of the relationship between inflation and export growth. This analysis is a crucial econometric tool aimed at examining how the relationship between variables shifts when a specific threshold value of another variable is crossed [27]. In the realm of export growth in COMESA, scholars implies that the influence of trade openness and economic growth on export performance exhibit variability contingent upon heterogynous levels of inflation [69]. A low, stable inflation environment might foster export growth by providing a predictable economic climate, encouraging investment, and reducing transaction costs [70,71]. Nevertheless, if inflation surpasses an optimal threshold, it may jeopardize economic stability, escalate production expenditures, erode purchasing power, disrupt supply chain, and subsequently impede export performance and competitiveness [70,72–74]. By determining an optimal threshold value for inflation, this study seeks to offer actionable insights into how economic stability shapes export performance among COMESA member countries [70,71]. Table 4 summarizes the results that reflects the key variables and their coefficients under different regimes.

**Table 4:** Threshold effects analysis.

Threshold effects analysis	
Parameter	Value
Minimum Residuals Sum of Squares (RSS)	28.552
Optimal Threshold (LnInfl)	3.662

The results of this analysis reveal an optimal threshold value for inflation, identified at 3.662%, and a corresponding minimum Residual Sum of Squares (RSS) of 28.552. These parameters are essential for evaluating the model’s goodness of fit. Moreover, our findings align closely with those of [55], who conducted an extensive study on inflation thresholds in the African context. Ndoricimpa’s research suggests that an inflation rate exceeding 5.43% negatively impacts economic growth, while maintaining inflation rates below this threshold does not exhibit detrimental effects. This correlation emphasizes the significance of identifying accurate inflation thresholds to inform economic policy and promote sustainable growth.

The comprehensive investigation conducted within the parameters of this study has ascertained that the optimal threshold inflation rate for COMESA during the extensive timeframe spanning from the year 2000–2022 has been identified as precisely 3.662%, a figure that holds significant implications for economic policy within the region. This particular inflation rate corresponds remarkably closely with the lower boundary of the inflation targets that are conventionally established in both emerging and developing economies, which typically fluctuate within a range of approximately 3% to 7%, thereby indicating a broader context for economic performance [75–77]. Furthermore, this identified inflation rate also occupies a position at the upper limit of the more conservative inflation range of 2% to 3% that is commonly targeted by central banking authorities in various developed economies, which collectively suggests that this figure represents a judicious and realistically attainable goal for the maintenance of economic stability throughout the COMESA region [75–77].

**Fig 3:**
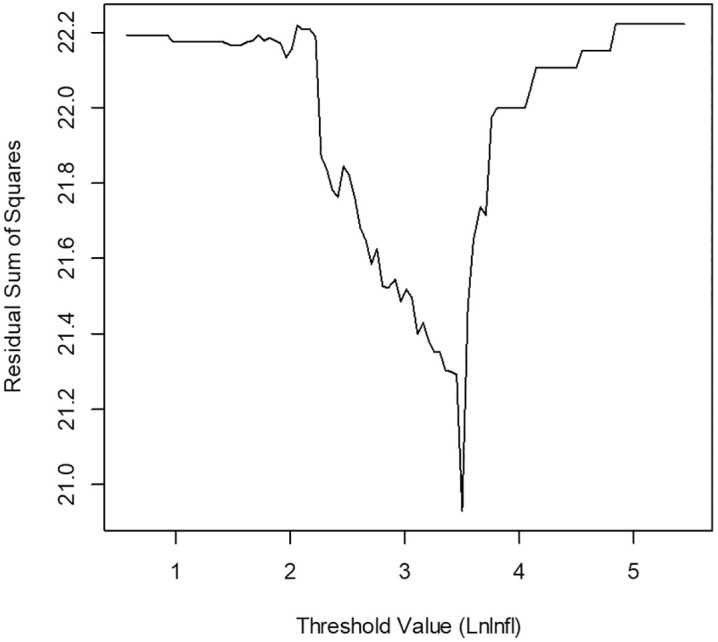
Residual sum of squares (RSS) vs. optimum threshold values for inflation.

Fig 3 illustrates how RSS changes across different potential threshold values of inflation (grid search) [57], with a clear minimum point at a threshold value of 3.662, indicating a structural break in the relationship between export growth and its predictors, suggesting that splitting the data into two regimes, low inflation (LnInfl≤3.662) and high inflation (LnInfl>3.662), at this point provides the best model fit by reducing prediction errors.

Generally, the threshold effects analysis reveals that inflation serves as a significant determinant in understanding the interplay between GDP, trade openness, and export growth in the COMESA region. The divergent effects in low- and high-inflation regimes underline the importance of maintaining economic stability to foster an environment where both GDP growth and trade openness can effectively contribute to boosting export performance.

### 4.3. Testing for nonlinear relationships

Scholars agree that testing for nonlinear relationships is imperative, given several economic theories posit that the effects of policies or economic conditions are not invariably consistent [69,78]. One of the key objectives of this analysis is to comprehend how sensitivity to economic conditions varies across different inflation levels. Through the integration of nonlinear specifications, this study seeks to enhance economic models, thereby providing a more robust framework that accurately encapsulates the complexities of these interrelations.

In our exploration of the relationship between export growth and its predictors (GDP, trade openness, and inflation), we sought to determine whether there is a nonlinear connection based on panel data during the study period (see Table 5). This inquiry highlights that the interactions among these factors may change under varying economic circumstances, particularly influenced by inflation rates. First, we compared the fit of a linear regression model against a quadratic regression model to see if including polynomial regression for inflation capture potential nonlinearities in the relationship and provided a better explanation of export growth [58].

**Table 5:** Nonlinearity testing using quadratic regression.

Model	Res. Df	RSS	Df	Sum of Sq	F	Pr(>F)
**Model 1**: (LnEG ~ LnInfl + LnGDP + LnTO)	272	22.214	–	–	–	–
**Model 2**: (LnEG ~ poly(LnInfl, 2) + LnGDP + LnTO)	271	22.213	1	0.001	0.011	0.013

The results from the ANOVA indicated that the quadratic model (Model 2) significantly outperform (Model 1), the traditional linear model (lm), obtaining an F-statistic of 0.011 with a *p*-value of 0.013. These results suggest that there is curvature in the relationship between inflation and export growth, supporting the hypothesis of nonlinearity [58]. The presence of nonlinear relationships suggests that the effects of GDP and trade openness on export growth might not be uniform; for example, a certain level of inflation could lead to disproportionately larger increases in exports as economic growth rises.

### 4.4. Panel data threshold regression (PTR)

By utilizing a PTR approach, we divided the data based on inflation levels, specifically focusing on a threshold defined by the median inflation rate. This division allowed us to analyze the impacts of GDP and trade openness in two distinct inflation regimes: low inflation and high inflation. The results of the PTR analysis for the low- and high-inflation regime, presented in Table 6, reveal significant relationships between export growth, GDP, and trade openness.

**Table 6:** Panel data threshold regression (PTR) for low- and high-inflation regime.

	Estimate	Std. Error	t value	Pr(>|t|)	Regime
LnGDP	0.348	0.057	6.023	1.80e-08 ***	Low inflation
LnTO	0.091	0.064	1.415	0.159	Low inflation
F-statistic	20.785			1.64e-08 ***	Low inflation
LnGDP	0.779	0.142	5.456	2.54e-07 ***	High inflation
LnTO	−0.127	0.062	−2.016	0.046 *	High inflation
F-statistic	16.148			5.85e-07 ***	High inflation

Significance: ‘***’ 0.001 ‘**’ 0.01 ‘*’ 0.05.

In the low-inflation regime, which includes observations where inflation is below the median threshold, our findings revealed that LnGDP has a strong positive effect on export growth, with a coefficient of 0.348 and a highly significant *p*-value (1.802e-08). This suggests that for every 1% increase in LnGDP, export growth experiences an approximate increase of 0.348%. Scholars like [79] provides evidence that supports our findings. He conducted a study on over 40 African countries from 1960 to 2018, using various econometric methods to investigate the relationship between export growth and GDP growth. The study found a positive, bidirectional relationship between exports and economic growth. This significant relationship underscores the importance of economic growth in fostering exports during periods of low inflation [79,80]. However, while trade openness also has a positive coefficient of 0.091, it is not statistically significant (*p* = 0.159). This indicates that although greater trade openness could contribute to higher exports, its effect is less pronounced in a low-inflation context. Furthermore, the insignificance of trade openness may reflect persistent structural challenges faced by COMESA economies, such as non-tariff barriers, inadequate infrastructure for cross-border trade, lack of logistics networks, regulatory bottlenecks, limited diversification of traded goods, and insufficient regional integration frameworks within COMESA. These issues may persist even in the presence of open trade policies, potentially leading to increased transaction costs, currency depreciation, and diminished competitiveness in global markets driven by price instability.

When we examined the high-inflation regime, where inflation exceeds the median threshold, the dynamics shifted substantially. Here, LnGDP remained significant, with an even larger positive coefficient of 0.779 (*p* = 2.538e-07), indicating that the impact of LnGDP on export growth is even stronger in times of high inflation. Specifically, a 1% increase in LnGDP corresponds to an impressive 0.779% increase in export growth. On the other hand, trade openness in this regime displayed a negative coefficient of −0.127, which is statistically significant (*p* = 0.046). This result is particularly striking as it suggests that during high-inflation periods, increased trade openness may actually hinder export growth. Our findings contradict the results of [39,81]. The results also highlight that the relationship between trade openness and export growth is not always positive. Some studies suggest the export-led growth hypothesis does not hold for all countries [11,82]. Other studies also confirm that the impact of trade openness can vary based on a country’s level of development and openness [36,83]. This unexpected finding could point to potential structural inefficiencies within COMESA economies that manifest during inflationary episodes. In terms of model performance, the F-statistic of both regimes and their associated *p*-value of < 0.001 confirm that the model as a whole is statistically significant, confirming their overall reliability.

**Fig 4:**
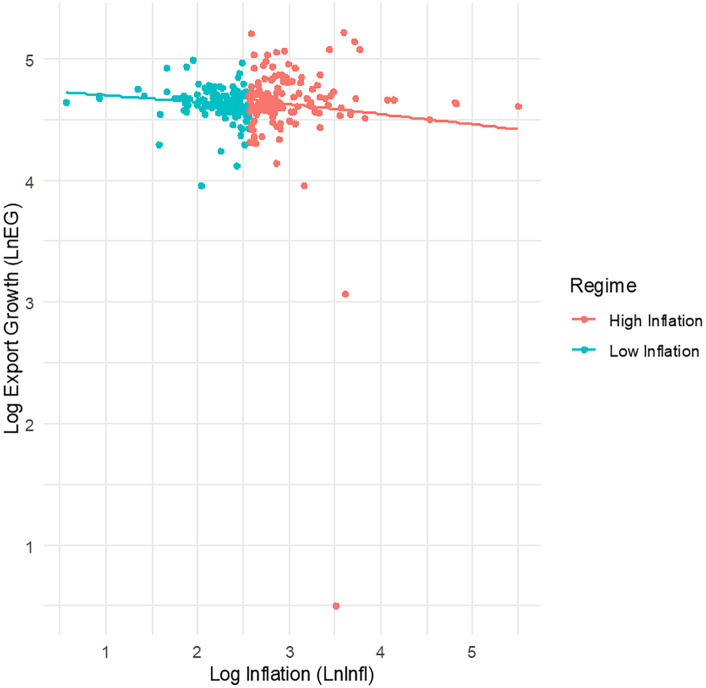
Export growth against inflation with fitted lines from both regimes.

To further illustrate how inflation interacts with export growth across different regimes, Fig 4 depicts the relationship between export growth and inflation, showcasing fitted lines from both models. By visually representing these dynamics, we can better grasp the nuanced effects of inflation on export growth in the context of COMESA economies.

Overall, our findings highlight that in the low inflation regime, maintaining economic stability through controlled inflation fosters an environment where economic growth significantly enhances export performance within COMESA countries during the study period. However, trade openness does not show a direct and significant impact on exports under these conditions, likely due to structural challenges or inefficiencies in policy implementation. In contrast, during high inflation periods, while economic expansion continues to support exports, increased trade openness may expose economies to risks that negatively affect export performance. To address these issues, policymakers should prioritize macroeconomic stability by implementing effective monetary and fiscal policies to control inflation. Additionally, investments in productivity-enhancing measures such as infrastructure development and technological advancements are essential to reduce production costs and improve competitiveness in global markets.

### 4.5. Interaction terms between inflation and other variables

In this section we investigated how inflation interacts with other key economic factors, namely GDP and trade openness, in affecting export growth within the COMESA region from 2000 to 2022. We included interaction terms in our regression model to capture these relationships more accurately, see Table 7. Our findings revealed several important insights. First, the model’s intercept, estimated at 4.794, indicates a strong baseline level of export growth when all other factors are held constant. This is statistically significant, reflecting meaningful export levels in the absence of other influences. When looking specifically at GDP, we found a coefficient of −0.203, suggesting a negative relationship with export growth; however, this result was not statistically significant, indicating that LnGDP alone might not consistently affect export performance in our model. In terms of trade openness, we observed a positive coefficient of 0.117, but, again, this was not statistically significant, indicating that while trade openness may positively influence exports, its effect is not strong enough to be conclusive. On the other hand, inflation had a notable negative effect with a coefficient of −0.722, which was statistically significant (*p* = 0.032). This suggests that rising inflation rates tend to decrease export growth, pointing to the detrimental impact of inflation on economic stability and competitiveness.

**Table 7:** Summary of interaction model results.

	Estimate	Std. Error	t-value	Pr(>|t|)
(Intercept)	4.794	1.037	4.622	5.9e-06 ***
LnGDP	−0.203	0.323	−0.630	0.529
LnTO	0.117	0.084	1.391	0.165
LnInfl	−0.722	0.334	−2.160	0.032 *
LnInfl_GDP	0.284	0.108	2.614	0.009 **
LnInfl_TO	−0.042	0.027	−1.562	0.119
F-statistic	13.720			6.03e-12 ***

Significance: ‘***’ 0.001 ‘**’ 0.01 ‘*’ 0.05.

A particularly interesting finding came from the interaction term between inflation and GDP (LnInfl_GDP), which showed a significant positive coefficient of 0.284 (*p* = 0.009). This result suggests that as GDP increases, the negative impact of inflation on export growth is decreased. Essentially, economic growth can help counterbalance some of the adverse effects of inflation on exports, highlighting the importance of fostering growth to stabilize trade outcomes. In the case of the interaction between inflation and trade openness (LnInfl_TO), the coefficient was −0.042. While this result was not statistically significant, it indicates a potential trend where the positive effects of trade openness on export performance might be diminished in high-inflation environments. This points to a complex interaction where higher LnGDP can potentially counterbalance the adverse effects of inflation on exports, evidenced by a statistically significant positive interaction term between inflation and LnGDP, while the interaction with trade openness remains less conclusive [2,84].

Economic growth has the potential to alleviate some of the detrimental effects of inflation on exports by enhancing productivity and bolstering international competitiveness [85]. Inflation can diminish a nation’s export competitiveness by elevating production costs and the prices of goods destined for international markets [86]. Nonetheless, economic growth can effectively counteract these adverse effects through several mechanisms.

First, economic growth typically facilitates advancements in technology and infrastructure, which can reduce production costs [87]. This increase in efficiency can mitigate the inflationary pressures on export prices, thereby rendering them more competitive on the global stage. Second, economic growth can lead to currency appreciation [88]. A stronger domestic currency enhances the attractiveness of exports for foreign purchasers by improving their purchasing power [89]. This dynamic can contribute to the maintenance or potential increase in export volumes even amidst inflationary challenges. Finally, economic growth can invigorate foreign demand for a country’s goods and services [90]. As economies worldwide expand, the demand for imports tends to rise, presenting exporting nations with greater opportunities to boost their sales [49].

The overall model fit is strong, as indicated by an F-statistic of 13.72 with a highly significant *p*-value (<6e-12), suggesting that the model explains a substantial portion of variation in export growth across COMESA countries during the study period. Generally, these results contribute to a nuanced understanding of how economic stability, particularly through inflation and GDP dynamics, influences export growth in the COMESA region. They emphasize the need for strategies focused on stabilizing inflation to create a more favorable environment for exports. Additionally, policymakers should prioritize boosting GDP, as economic growth can help mitigate some of the challenges posed by inflation.

## 5. Conclusion

This study contributes to the extant body of literature: *First*, by providing an exhaustive examination of the nonlinear threshold dynamics of inflation concerning export expansion, underscoring that the preservation of economic stability within low-inflation environments can amplify the advantages derived from GDP growth and trade liberalization. *Second*, through the application of the rigorous PTR methodology, this investigation delineates particular inflationary regimes and elucidates their discrete effects on export advancement. Such a methodology facilitates a more nuanced understanding of the intricacies and disparities in the effects of GDP and trade openness on exports under varying inflationary scenarios, thereby contesting conventional perspectives on these interrelationships. *Third*, this study addresses a significant gap in the existing literature by illustrating how nonlinear relationships between macroeconomic variables shape trade outcomes in developing regions like COMESA, contributing to a more nuanced investigation of nonlinear threshold effects beyond traditional linear analyses.

Economic stability plays a crucial role in determining export performance, especially in regions like the COMESA, where economies are often susceptible to external shocks, inflationary pressures, and fluctuating trade policies. Despite ongoing efforts to promote trade integration and economic growth within COMESA, there is limited empirical evidence on how inflation thresholds influence the relationship between GDP, trade openness, and export growth. This study aims to address this empirical gap by analyzing the nonlinear effects of inflation on export growth using panel data from 2000 to 2022.

The study identified an optimal threshold inflation rate of 3.662% for COMESA during the period from 2000 to 2022. This statistic is in close proximity to the lower spectrum of inflation target commonly observed in emerging and developing economies, which typically fluctuate between 3% and 7% [75–77]. Furthermore, it resides at the upper boundary of the 2–3% target range established by central banking authorities in numerous developed nations, indicating a judicious and attainable benchmark for sustaining economic stability within the COMESA region [75–77].

This study investigates threshold effects and evaluates nonlinear relationships, aiming to offer a more precise and policy-relevant understanding of the determinants of export performance in COMESA [68,91]. By acknowledging that the influences of economic growth and trade openness on export performance may vary considerably beyond certain inflationary thresholds (3.662%), the study can deliver articulated perspectives on optimal policy measures. This methodology transcends the constraints of linear models, effectively portraying the complex, dynamic, and frequently non-proportional characteristics of economic relationships, which is critical for effective economic policymaking aimed at promoting sustainable export growth [67,68,78,91]. Our findings emphasize the critical need for macroeconomic stability to promote sustainable export growth in COMESA member states. Policymakers should prioritize keeping inflation below the optimal threshold of 3.662%, as this is essential for maximizing the positive impacts of GDP growth and trade openness on export performance [10]. For countries experiencing high inflation, targeted interventions are necessary to stabilize prices and implement structural reforms that enhance productivity and competitiveness.

The PTR analysis reveals that in a low-inflation regime, GDP significantly boosts export growth by approximately 0.348% for each 1% rise in GDP, while trade openness shows no significant impact. Scholars regarding Botswana [92] and Nepal [2] corroborated a similar idea. Conversely, in a high-inflation regime, GDP’s effect on export growth intensifies to 0.779% per 1% increase, and trade openness negatively affects exports (0.127%), indicating a potential slowdown due to structural inefficiencies in the economies analyzed [81,93]. Overall, the PTR analysis findings emphasize the critical role inflation plays in shaping the effects of economic growth and trade openness on export growth, highlighting distinctly different impacts in low- versus high-inflation scenarios [94]. This transition from a positive to a negative impact of trade openness underscores how macroeconomic instability, evidenced by high inflation, can weaken the benefits associated with trade liberalization. This finding aligns with economic theory suggesting that high levels of inflation create uncertainty and distortions in international trade markets by increasing transaction costs and reducing competitiveness [94–96]. To emphasize more, the diverse effects of inflation play a significant role in shaping the relationship among economic growth, trade openness, and the expansion of exports by influencing price competitiveness, the distribution of resources, and overall economic stability. High inflation rates may reduce a nation’s export price competitiveness, making its goods less appealing in international markets [94]. On the other hand, moderate inflation might indicate a robust economy with rising demand, potentially encouraging higher production levels for exports [94].

Notably, the interaction term between inflation and GDP (LnInfl_GDP) showed a significant positive coefficient of 0.284, suggesting that higher GDP can mitigate the negative impact of inflation on exports. In contrast, the interaction between inflation and trade openness (LnInfl_TO) indicated a potential diminishing effect on export performance, though it was not statistically significant [2,84]. Inflation demonstrated a significant negative effect on export growth with a coefficient of −0.722. Scholars contend that inflation significantly undermines export performance [85]. Inflation can diminish a nation’s export competitiveness by elevating production costs and the prices of goods destined for international markets [86]. Several studies support the idea that managing inflation and promoting economic growth are crucial for stabilizing trade outcomes. Overall, both models are statistically significant with an F-statistic *p*-value of <0.001.

## 6. Policy recommendations and future research direction

In light of the findings presented in this study, we propose the following policy recommendations to enhance export performance within the COMESA: To enhance exports in the COMESA, policy interventions must focus on macroeconomic stability, infrastructure development, financial sector reforms, and regional integration. Firstly, stabilizing macroeconomic frameworks is vital for export competitiveness; policies should aim to control inflation, maintain stable exchange rates, and create a conducive business environment as these factors significantly influence a nation’s international market position. Secondly, investing in infrastructure, especially transportation and digital connectivity, and diversifying export markets are crucial for reducing trade costs, enhancing logistics efficiency, and mitigating the adverse threshold effects of economic instability. Additionally, fostering financial sector reforms to improve credit access is essential, particularly for small and medium enterprises (SMEs) that often struggle with financing their export activities [97–99]. Promoting regional integration through harmonizing trade regulations, reducing tariffs, and simplifying customs procedures will further boost exports within COMESA. Furthermore, regional integration strategies should address existing macroeconomic disparities that hamper export competitiveness, highlighting the need for a comprehensive approach to foster economic resilience [4].

This study has certain limitations. Firstly, it relied on panel data from 2000 to 2022, which may not fully capture short-term shocks or structural changes impacting individual COMESA countries. Secondly, factors such as exchange rate volatility, political stability, and external demand conditions were not included, even though they could significantly influence export performance. Lastly, while the focus was on COMESA nations, the findings may not be applicable to other regions with differing economic structures and policy environments.

Future research should expand this model by including additional macroeconomic variables, such as exchange rate volatility, FDI, political stability, and institutional quality, to deepen the understanding of export influences. Comparative studies across other regional economic blocs like ECOWAS, SADC, or East African Community (EAC) could determine if similar inflation thresholds apply under different economic contexts. Utilizing quarterly data instead of annual data may capture short-term dynamics more effectively, while sector-specific analyses could reveal how inflation thresholds impact various industries differently. Investigating asymmetric effects, particularly whether reductions in inflation yield proportional improvements in exports, would also be beneficial. Furthermore, future research should delve into country-specific impacts and causal mechanisms linked to economic stability, the effects of external shocks on export performance, and the role of digital trade facilitation in enhancing market access. Exploring how government size and institutional quality affect export competitiveness is also critical for formulating effective policies.

## Supporting information

S1 Table Summary of COMESA member countries excluded from the study due to insufficient data.
(DOCX)

S2 Data This Excel file contains the log-transformed values for all key variables examined in the study, including export growth (LnEG), economic growth (LnGDP), trade openness (LnTO), and inflation rate (LnInfl). These transformations facilitate a more robust statistical analysis and interpretation of the relationships among these variables. Additionally, the file presents comprehensive empirical results that underpin the findings discussed in the main manuscript. Key components include: • Descriptive Statistics: Summary statistics for variables across low- and high-inflation regimes, providing insights into the data distribution and variance. • Threshold Effects Analysis: An investigation into how the relationship between variables changes across different inflation levels. • Nonlinearity Testing: Results from quadratic regression analyses to assess nonlinear relationships among the variables. • Panel Data Threshold Regression (PTR): Detailed results of the PTR model for both low- and high-inflation regimes, highlighting the differential impacts in varying economic contexts. • Interaction Model Results: A summary of findings from interaction models that explore the interplay between key variables across inflation regimes. Moreover, this file includes additional empirical findings that, while not presented in the main text, are significant for enhancing understanding and guiding further investigations. This supporting information is designed to complement the narrative results in the manuscript, providing further context and depth to the interpretations discussed.
(XLSX)
