# Supplementary material for: The role of economic stability in boosting exports in COMESA: Threshold effects analysis
Source: PLoS One. 2025 Dec 11;20(12):e0338636. doi: 10.1371/journal.pone.0338636 (PMC12697952; doi:10.1371/journal.pone.0338636)
Supplement: S1 Table — Summary of COMESA member countries excluded from the study due to insufficient data. (DOCX) [file pone.0338636.s001.docx]

**S1 Table**

Summary of COMESA member countries excluded from the study due to insufficient data

| **Country** | **LnEG** | **LnFDI** | **LnGDP** | **LnTO** | **LnInfl** |
| --- | --- | --- | --- | --- | --- |
| DRC | N/A (1995-2010) |  |  |  |  |
| Djibouti | N/A (1995-2013) |  | N/A (1995-2014) |  |  |
| Eritrea | N/A (2012-2022) | N/A (2012-2022) | N/A (2012-2022) | N/A (2012-2022) | N/A (2012-2022) |
| Ethiopia | N/A (1995-2011) |  |  | N/A (1995-2010) |  |
| Libya | N/A (1995-2003) | N/A (2014-2022) | N/A (1995-2000) |  | N/A (1995-1999) |
| Malawi | N/A(1995-2022) |  |  |  |  |
| Somalia | N/A (1995-2022) | N/A (1995-2012) | N/A (1995-2014) |  | N/A (1995-2012) |
| Zambia | N/A (2011-2022) |  |  |  |  |
| Zimbabwe | N/A (1995-2004) |  |  |  |  |

Note: Data not available (N/A), Democratic Republic of the Congo (DRC)
